# Drug-Induced Eosinophilic Pneumonia as an Adverse Event of Abemaciclib

**DOI:** 10.7759/cureus.21741

**Published:** 2022-01-30

**Authors:** Yuki Mitarai, Yukari Tsubata, Miki Hyakudomi, Takeshi Isobe

**Affiliations:** 1 Department of Respiratory Medicine, National Hospital Organization Hamada Medical Center, Hamada, JPN; 2 Department of Internal Medicine, Division of Medical Oncology & Respiratory Medicine, Shimane University Faculty of Medicine, Izumo, JPN; 3 Breast Surgery, Hyakudomi Clinic, Izumo, JPN

**Keywords:** eosinophil, breast cancer, abemaciclib, eosinophilic pneumonia, drug-induced lung injury

## Abstract

In 2020, a 45-year-old woman was started on fulvestrant and abemaciclib therapy to treat breast cancer which had recurred in her left breast after surgery. We were able to control her cancer using this treatment; however, the ground-glass opacity in the lower lobe of her right lung expanded, along with an increase in her peripheral blood eosinophil count. She was referred to the respiratory medicine department for a detailed examination including bronchoscopy. We discovered a high proportion of eosinophils in her bronchoalveolar lavage fluid and diagnosed the condition as drug-induced eosinophilic pneumonia. The ground-glass change improved after steroid administration. In this case, the adverse effects of abemaciclib, a cyclin-dependent kinase 4/6 inhibitor playing an essential role in breast cancer treatment, were discovered by combining blood, imaging, and bronchoalveolar lavage fluid findings. This contributed to an early introduction of treatment and prevented the deterioration of her quality of life.

## Introduction

The incidence of drug-induced lung injury due to the cyclin-dependent kinase 4/6 (CDK4/6) inhibitor abemaciclib has been reported to be 2.1% [1], and it has been shown to become severe at times [2]. CDK4/6 inhibitors are key drugs used in breast cancer treatment. A clinical trial has shown that adding abemaciclib to postoperative endocrine therapy improves prognosis in hormone receptor-positive, human epidermal growth factor receptor 2 (HER2)-negative metastasis or recurrent breast cancer [3]. The Japanese Breast Cancer Practice Guidelines strongly recommend the combination of an aromatase inhibitor and a CDK4/6 inhibitor as primary endocrine therapy for postmenopausal hormone receptor-positive metastasis or recurrent breast cancer [4]. Therefore, to improve the prognosis of patients and continue treatment for breast cancer, it is necessary to detect and treat drug-induced pneumonia caused by abemaciclib at an early stage.

## Case presentation

**Figure 1:**
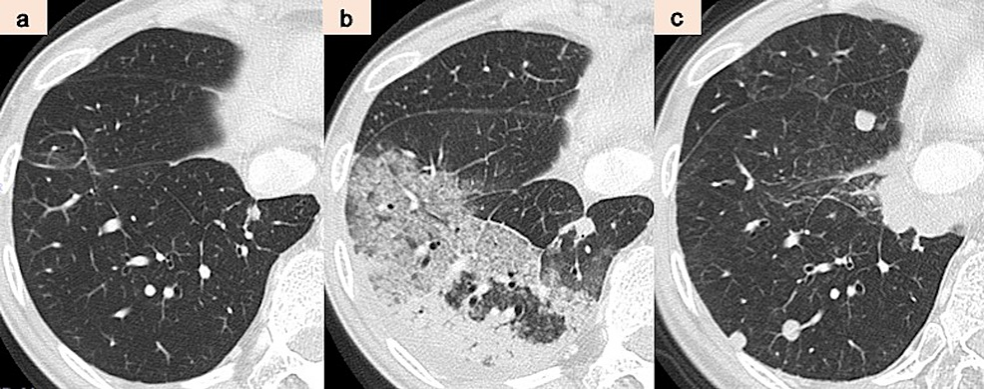
(a-c) Imaging findings on CT. CT: computed tomography

**Figure 2:**
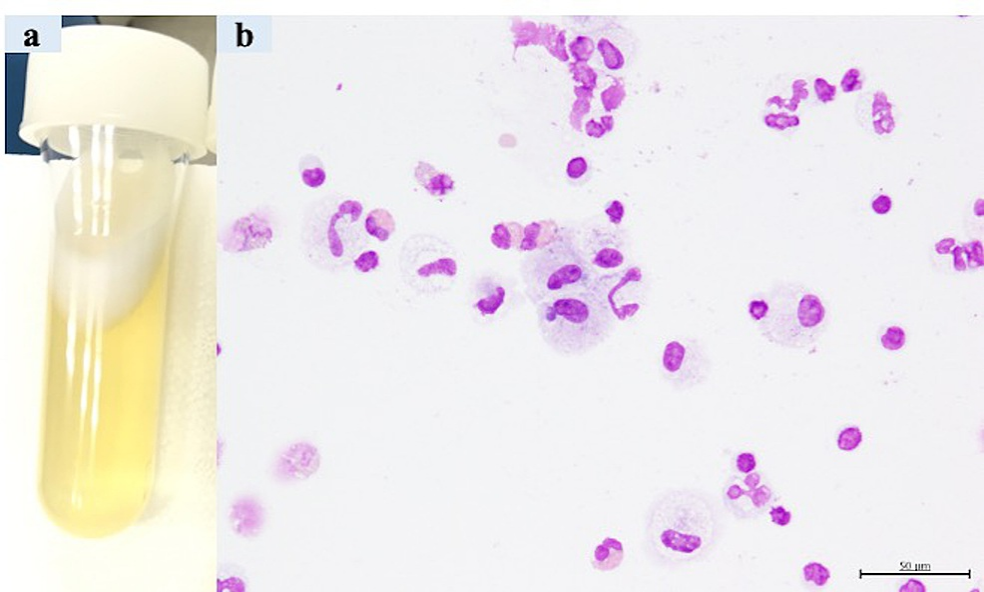
(a, b) Findings of the bronchoalveolar lavage fluid.

Our patient was a female with no medical history, no smoking history, and no occupational history, such as dust exposure, which could be associated with respiratory illness. In 2016, at the age of 40, she was diagnosed with breast cancer (adenocarcinoma, pT2N3aM0, stage IIIC, estrogen receptor (ER)-positive, and HER2+) and underwent a left mastectomy and axillary lymph node dissection at our hospital’s Breast and Endocrine Surgery Department. After receiving four courses of epirubicin and cyclophosphamide as adjuvant chemotherapy, she was treated with four courses of docetaxel and trastuzumab, 14 courses of tamoxifen and trastuzumab, and radiation therapy. At that time, there were no findings suggestive of radioactive pneumonitis in her lungs (Figure 1a). However, three months after the therapy, due to the appearance of lung metastasis, she received 14 courses of trastuzumab, pertuzumab, and docetaxel. Nevertheless, as imaging suggested metastasis to the lungs, she received the following chemotherapy: six courses of trastuzumab emtansine; six courses of trastuzumab, pertuzumab, and eribulin; and 11 courses of bevacizumab and paclitaxel. Subsequently, liver metastasis appeared in September 2019. On liver biopsy in 2020, while ER remained positive, we found HER2 expression to be negative. Therefore, fulvestrant and abemaciclib therapy was started after liver biopsy. Although the combination therapy controlled breast cancer progression, including metastasis to the liver and lung, she developed a mild cough and her peripheral blood eosinophil ratio increased to 13.9% after two weeks of administration of abemaciclib. Simultaneously, a frosted glass shadow appeared in the lower lobe of the patient’s right lung on chest CT (Figure 1b). Initially, she was diagnosed with bacterial pneumonia and given an antibacterial drug, but she was referred to our hospital’s Respiratory Medicine Department because her chest CT showed exacerbation of the ground-glass opacity. At the time of consultation with the Respiratory Medicine Department, the peripheral blood eosinophil ratio had increased to 22.1%. We performed a bronchoscopy and bronchoalveolar lavage (BAL) and collected a pale yellow transparent bronchoalveolar lavage fluid (BALF) (Figure 2a). The eosinophil proportion in BALF was high at 18% (Figure 2b). Based on the imaging findings, BALF findings, and high peripheral blood eosinophil levels, we established abemaciclib as the drug that induced eosinophilic pneumonia. After diagnosis, administration of steroid (prednisolone 30 mg/day) was started as anti-inflammatory therapy. After starting the treatment, the apparent disappearance of ground-glass shadows was noted (Figure 1c). Hence, we decided to gradually reduce the dose of prednisolone to 5 mg every two to three weeks. We terminated the steroid therapy after four months. Eosinophilic pneumonia has not recurred after initiating steroids, and, currently, resumption of treatment for breast cancer is being considered without abemaciclib.

## Discussion

We report a case of drug-induced eosinophilic pneumonia that was thought to be caused by abemaciclib. Interstitial lung disease, as a side effect of abemaciclib, has various imaging findings such as diffuse alveolar damage pattern, organizing pneumonia pattern, faint ground-glass opacity pattern, and non-specific interstitial pneumonia pattern [5]. In this case, widespread infiltration shadows were observed, and it was necessary to distinguish them from cryptogenic organizing pneumonia. Other non-tuberculous mycobacteriosis and fungal infections were also considered but were excluded because no significant findings were obtained on blood biochemical tests and BALF culture tests. In this case, eosinophilic pneumonia occurred after the administration of abemaciclib, and a clear improvement was noted on discontinuation of abemaciclib and introduction of steroids. Moreover, there was no record of the introduction of any new drug other than abemaciclib. In addition, no history of allergies was reported by the patient. Therefore, regarding the causal relationship between abemaciclib and eosinophilic pneumonia, the Naranjo score was 5 points, which was judged to be “probable” [6]. Therefore, we concluded that it was eosinophilic pneumonia caused by abemaciclib.

Both acute and chronic types of drug-induced eosinophilic pneumonia have been reported. This case was considered to be chronic eosinophilic pneumonia (CEP) that worsened after three months or more. A characteristic of CEP is an increase in the ratio of peripheral blood eosinophils, which can be an important basis for diagnosis [7].

In this case, the proportion of eosinophils in BALF as well as in blood was increased. It has been reported that BAL is indispensable for the diagnosis of drug-induced acute eosinophilic pneumonia and that the need for tissue biopsy is low if a diagnosis by BALF is obtained [8]. Because the symptoms were minor in this case, bronchoscopy could be performed without causing deterioration of the respiratory condition, and diagnosis by BAL was possible. Because there are no reports of BAL analysis for drug-induced interstitial pneumonia caused by abemaciclib, this case is valuable.

## Conclusions

In this case, proper diagnosis and introduction of steroid treatment could prevent the exacerbation of eosinophilic pneumonia and deterioration of the patient’s quality of life. To obtain a definitive diagnosis of drug-induced interstitial pneumonia by bronchoscopy, it is desirable to perform the examination after early detection. When administering abemaciclib, breast surgery and respiratory medicine are linked, and focusing on peripheral blood eosinophil ratio as well as imaging findings can lead to early detection of drug-induced eosinophilic pneumonia.
